# The Potential Link between Gut Microbiota and Serum TRAb in Chinese Patients with Severe and Active Graves' Orbitopathy

**DOI:** 10.1155/2019/9736968

**Published:** 2019-12-18

**Authors:** Ting-Ting Shi, Lin Hua, Hua Wang, Zhong Xin

**Affiliations:** ^1^Department of Endocrinology, Beijing Tongren Hospital, Capital Medical University, Beijing, China; ^2^Department of Mathematics, School of Biomedical Engineering, Capital Medical University, Beijing, China; ^3^Department of Emergency, Beijing Tongren Hospital, Capital Medical University, Beijing, China

## Abstract

**Background and Objective:**

A previous study reported alterations in the intestinal microbiota in patients with Graves' orbitopathy (GO). Thyrotropin receptor autoantibody (TRAb) stimulates orbital and periorbital tissues and plays a pivotal role in the development of GO. However, the association between gut microbiota and TRAb in GO patients has still remained elusive. In this study, we explored the relationships between gut microbiota and GO-related traits, in which we applied a metabolic-network-driven analysis to identify GO trait-related modules and extracted significant operational taxonomic units (OTUs).

**Methods:**

In the present study, we profiled gut microbiota using 16S rRNA gene sequencing in 31 GO patients. We performed metabolic-network-driven analysis to investigate the association between gut microbiota and GO-related traits (e.g., TRAb, TGAb, and TPOAb) in the combination of microbial effects.

**Results:**

Applying microbiome network analysis of cooccurrence patterns and analysis of topological properties, we found that *s_Prevotella_copri* and *f_Prevotellaceae* showed a significant correlation with TRAb. In particular, we applied the latent class model to explore the association between gut microbiota and GO-related traits in the combination of microbial effects. It was revealed that the subjects involved in the latent class model with the higher abundance of *s_Prevotella_copri* and *g_Bacteroides* had a higher TRAb level.

**Conclusions:**

Our results revealed the potential relationships between gut microbiota and GO-related traits in the combination of microbial effects. This study may provide a new insight into the interaction between the intestinal microbiota and TRAb-associated immune responses in GO patients.

## 1. Introduction

Graves' orbitopathy (GO) is an autoimmune disease, commonly associated with Graves' disease (GD). It influences appearance, visual acuity, and even quality of life of patients [1–3]. To date, the pathogenesis of GO has not been fully understood. Gut microbiota influences various autoimmune diseases, such as type 1 diabetes (T1D) [4] and systemic lupus erythematosus (SLE) [5]. A recently conducted study demonstrated that gut microbiota is associated with some thyroid diseases, including Hashimoto's thyroiditis (HT) and GD [6]. Increased intestinal permeability and infiltration of intraepithelial lymphocytes have been previously found in patients with HT [7]. Intestinal symbiotic microorganisms may influence extraintestinal immune responses inducing loss of tolerance to self-antigens, including thyroglobulin that underlies HT [8]. Our previous study revealed alterations in the intestinal microbiota in patients with severe and active GO. For instance, community diversity was significantly reduced in patients with GO. At the phylum level, the proportion of Bacteroidetes was notably increased, while at the genus and species levels, significant differences were observed [9]. The present study highlighted the role of microbiota in occurrence of GO.

To our knowledge, evaluating the autoimmune inflammation of GO patients into different stages, active and nonactive forms, is highly significant for the treatment of GO. An evidence showed that thyrotropin receptor autoantibody (TRAb) stimulates orbital and periorbital tissues and also plays a pivotal role in the development of GO; thus, detection of TRAb may be of clinical significance in active assessment of disease [10, 11]. Elevated expression of the thyroid-stimulating hormone (TSH) receptor in orbital tissues supports the substantial role of TRAb in the pathogenesis of GO [10, 12]. Recently, Kahaly et al. reported that high titers of TRAb are related to thyroid-associated orbitopathy in patients with GD [13]. However, to date, a limited number of studies explored the role of gut microbiota in GO patients, while none of the studies reported a potential association between gut microbiota and TRAb in such patients.

In the present research, to explore the relationships between gut microbiota and GO-related traits, e.g., TRAb and TGAb, we applied a metabolic-network-driven analysis to identify GO trait-related modules and extract important operational taxonomic units (OTUs). We identified some novel associations between gut microbiota and GO-associated traits. Our study provided a framework to better perceive the interactions of gut microbiota and extracted the important bacteria associated with TRAb.

## 2. Study Subjects and Methods

### 2.1. Study Subjects

In the current study, the 16S rRNA gene sequence was used to reconstruct the taxonomic structure of gut microbial communities using the fecal samples of GO patients. This study was performed at the Department of Endocrinology, Beijing Tongren Hospital, Capital Medical University, Beijing, China. Between March 2017 and March 2018, 31 patients with severe and active GO with hyperthyroid were enrolled. All patients received only an antithyroid drug (Thyrozol; Merck & Co., Inc., Kenilworth, NJ, USA). The diagnosis of GO was carried out according to the European Group on Graves' Orbitopathy (EUGOGO) guidelines [2]. The enrolled GO patients had not received any treatment for ocular discomfort. The active GO was defined by a clinical activity score (CAS) ≥3/7, and severe GO was defined by the NOSPECS score ≥IV. TRAb was measured using a commercially available electrochemiluminescence assay kit (Roche Diagnostics GmbH, Mannheim, Germany) based on the M22 monoclonal antibody, with a normal range <1.75 U/L. The exclusion criteria for the GO patients were patients' age <18 or >65 years, history of chronic diarrhea or constipation, history of gastrointestinal surgery, therapy of probiotics or antibiotics over the previous 4 weeks, use of hormonal medication (<3 months), severe disease (acute infections, diabetes, stroke, renal or hepatic dysfunction, cancer, or autoimmune diseases), pure vegetarian, etc. [9]. The characteristics of the study subjects are summarized in Table 1.

This study was approved by the Ethics Committee of Beijing Tongren Hospital, Capital Medical University (Registration No. TRECKY2016-003). Written informed consent was obtained from all the patients as well.

### 2.2. Stool Sample Microbiota Sequencing, OTU Cluster, and Species Annotation

In brief, approximately 2.5 g of a fresh fecal sample was collected from each participant using a plastic tube prefilled with the stool DNA stabilizer including a measuring spoon for sample collection (PSP Spin Stool DNA Plus Kit; STRATEC Molecular, Berlin, Germany). Bacterial DNA from fecal samples was extracted according to the manufacturer's instructions (PSP Spin Stool DNA Plus Kit; STRATEC Molecular). DNA concentrations were assayed using a NanoDrop 2000 Bioanalyzer at 260 nm (Thermo Fisher Scientific Inc., Waltham, MA, USA). 16S rRNA genes of distinct regions (16S V4/16S, 515F: GTGCCAGCMGCCGCGGTAA; 806R: GGACTACHVGGGTWTCTAAT) were amplified using specific primers [14].

The sequences were analyzed and those with ≥97% similarity were classified into the same OTUs. The representative sequence for each OTU was screened out for further annotation. The abundance information on the OTUs was normalized using a standard sequence number, corresponding to each sample with the least number of sequences as previously described [9].

### 2.3. Network Analysis

#### 2.3.1. Cooccurrence Network

The composition of bacterial communities could be positively influenced, in addition to negative relationships between contributing microorganisms in human disease. Thus, intermicrobial relationships can be inferred from the cooccurrence network of taxa, and this network can be used to investigate ecological interactions between microbes [15]. In the present research, we identified cooccurrence networks of gut microbiota based on Spearman's correlation analysis using the relative abundance tables, and *P* values were adjusted for multiple testing using the Benjamini–Hochberg approach. These networks kept those correlations with the adjusted *P* value <0.1, and the absolute value of correlation coefficients was >0.3. By applying the modularity scores, we attempted to find out a dense subgraph. We applied the igraph package of R software (http://www.r-project.org) to implement the analysis.

#### 2.3.2. Weighted Gene Coexpression Network Analysis (WGCNA)

Herein, the WGCNA was used to generate the network and identify network modules based on the OTU relative abundance. In WGCNA, we kept those OTUs that were found in at least 30% of the samples in order to ensure that the data were less sparse, as well as being more amenable to calculate correlation coefficients. In addition, as data dimensionality reduction is essential for the limited sample size, we referenced a previous study for preselecting bacteria. Eventually, the relative abundance of 51 OTUs was applied for WGCNA. Module preservation was assessed using the Z-summary as implemented in the WGCNA package of R software. We, in the present study, selected three topological properties (degree, betweenness, and closeness) of the network to extract important OTUs from the network. Accordingly, those OTUs with higher degree, betweenness, and closeness demonstrate that those are potential OTUs associated with GO.

#### 2.3.3. Correlation between GO-Related Traits and Gut Microbiota

We performed the correlation analysis between the modules and GO-related traits, which included TRAb, TPOAb, TGAb, and CAS. In the module-trait correlation analysis, the module eigenOTU was defined as the first principle component of a module, which was used to calculate Pearson's correlation coefficient between a module and a GO-related trait. Significance of the correlation was determined by an asymptotic *P* value. For those modules associated with GO-related traits, Pearson's correlation analysis was carried out to determine the association between each of the OTUs involved in the modules and GO-related traits.

#### 2.3.4. Negative Binomial Regression Model for Further Investigation of Relationships between GO-Related Traits and Gut Microbiota

As the identified bacteria showed a significant association with GO-related traits based on Pearson's correlation analysis, we further investigated the relationships between these bacteria and GO-related traits after adjusting for age and sex. Owing to the skewed and overdispersed distribution of the absolute abundance of bacterial taxa, the negative binomial regression model was used in the analysis. The absolute abundance of each microbiota was taken as a dependent variable, while TRAb, TPOAb, TGAb, and CAS were taken as independent variables, and age and sex were adjusting factors. *P* < 0.05 was considered statistically significant.

#### 2.3.5. Latent Class Analysis (LCA) to Classify GO Patients

**Figure 1:**
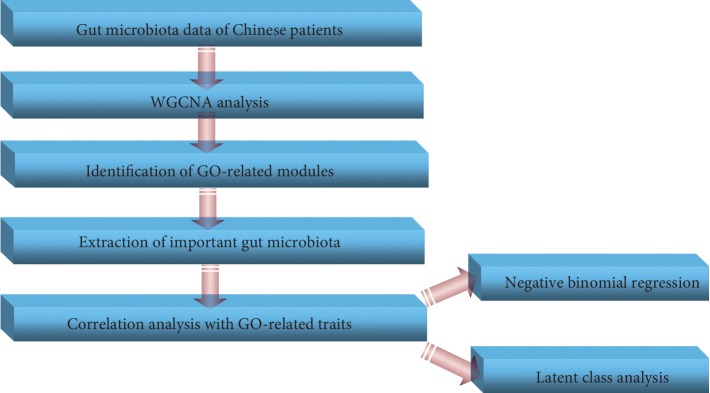
Flowchart of our work. Firstly, WGCNA was used to generate the network and to identify GO-related network modules based on the OTU relative abundance. Those gut microbiota with outstanding topological properties were extracted. Secondly, the module-trait association analysis was performed, and the Pearson correlation analysis was used to determine the association between each of the OTUs involved in the modules and GO-related traits. Thirdly, the relationships between the identified bacteria and GO-related traits were further investigated based on the negative binomial regression after adjusting for age and sex. Finally, the identified bacteria were used to classify GO patients by applying the latent class analysis.

As the identified bacteria showed significant association with GO-related traits, we further used them to classify GO patients by applying LCA and indicate their contributions to GO-related traits. LCA is a subset of structural equation modeling and is used to find out groups or subtypes of cases in multivariate categorical data, in which these subtypes were previously called “latent classes” [16]. At present, the latent class models are rarely applied in the analysis of microbiome data, in spite of the evolutionary, temporal, and count structure that could be directly incorporated through such models [17]. In the current study, we tried to use LCA to analyze the microbiome data: the absolute abundance of the identified bacteria was divided into three levels (<33rd percentile, >33rd percentile and <66th percentile, and >66th percentile), and these three levels were encoded as 1, 2, and 3. The LCA can detect the presence of the combination patterns for identified bacteria in latent classes. The general evaluation indexes of LCA are as follows: likelihood ratio (LR), Akaike information criterion (AIC), Bayesian information criterion (BIC), and entropy. The smaller the LR, the better the model fits the data. The smaller values of the AIC and BIC indicate the better fitting of the data. The greater the entropy, the better the model fits the data. Herein, we applied multiple evaluation indexes which outperformed the single index to determine the number of latent classes. To our knowledge, different indexes may have different evaluation results for the model; thus, the interpretation of the model is extremely important. We also compared differences in CAS, TRAb, TPOAb, and TGAb among the latent classes. We attempted to indicate whether gut microbiota contribute to the GO-related traits. The flowchart of our study is shown in Figure 1.

## 3. Results

### 3.1. Cooccurrence Network of Gut Microbiota

**Figure 2:**
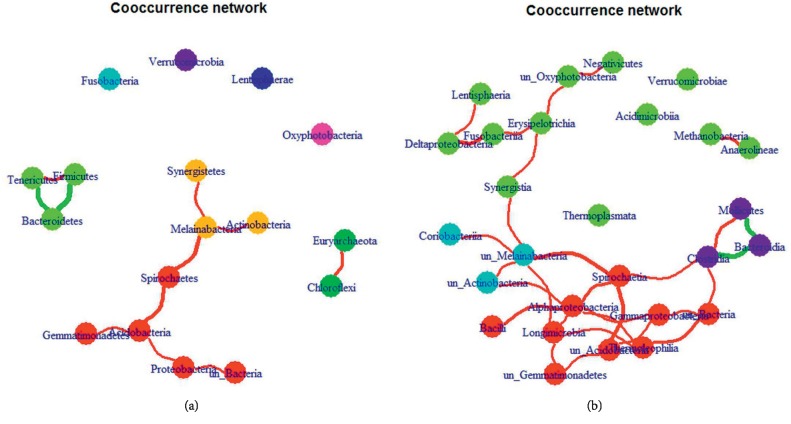
Operational taxonomic unit- (OTU-) based cooccurrence network analyses. The cooccurrence network of gut microbiota was constructed based on the correlation analysis. By applying the modularity scores, the dense communities were identified in graphs at the (a) phylum level and (b) class level.

In the present research, Pearson's correlation analysis was used to quantify the cooccurrence network of gut microbiota. High correlation reflected the interactions between sources of bacteria and similarities in their responses to environmental conditions. By applying the modularity scores, we found the dense communities in graphs. At the phylum level, the number of positive correlations was 9, whereas the number of negative correlations was 2, and the number of modalities was 3 (Figure 2(a)). As illustrated in Figure 2(a), Fimicutes and Bacteroidetes, as the most predominant phyla in GO patients, are involved in the same module. At the class level, the number of positive correlations was 28, whereas the number of negative correlations was 2, and the number of modalities was 4 (Figure 2(b)).

### 3.2. WGCNA

In WGCNA, we set up deepSplit = 2 and minModuleSize = 10 as parameters for the dynamic tree cut algorithm. The soft thresholding of power can be used in network construction; thus, we selected power = 1, which caused the fitted *R*^2^ value and the mean connectivity to be the highest (Supplementary [Supplementary-material supplementary-material-1]).

**Figure 3:**
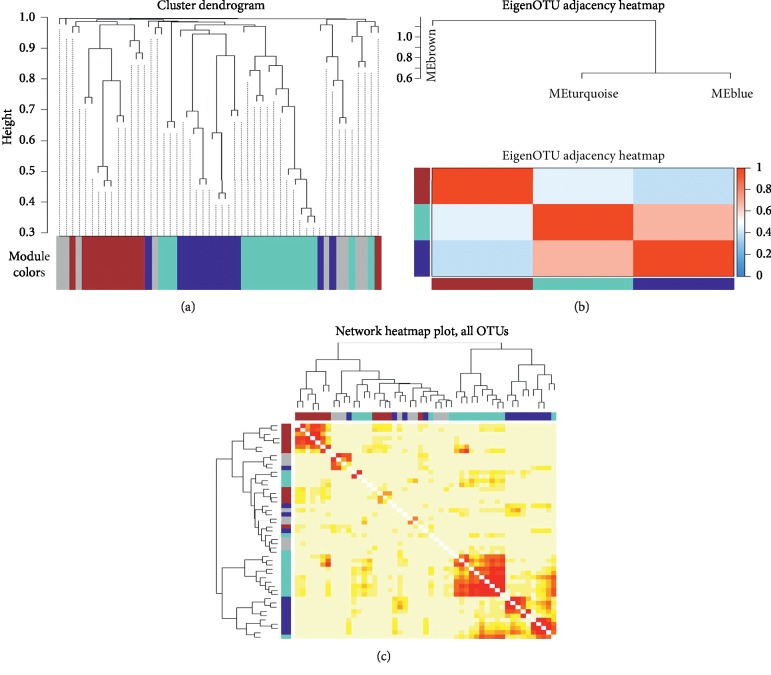
Identified modules by WGCNA. (a) Cluster dendrogram of four identified modules. (b) Adjacency heatmap of eigenOTU which is defined as the first principle component of a module. (c) Tom graph (network heatmap of all OTUs).

Four modules, namely, MEgrey, MEturquoise, MEblue, and MEbrown, were eventually selected. Meanwhile, MEgrey was taken as the reference module into account, which included 9 OTUs, and the number of OTUs in MEturquoise, MEblue, and MEbrown was 17, 13, and 12, respectively. MEgrey, MEturquoise, MEblue, and MEbrown were colored grey, turquoise, blue, and brown, respectively. The cluster dendrogram of four identified modules is displayed in Figure 3(a).  Figure 3(b) depicts the heatmap of an adjacency matrix of eigenOTU, which was defined as the first principle component of a module. The network heatmap of all OTUs is shown in Figure 3(c).

**Figure 4:**
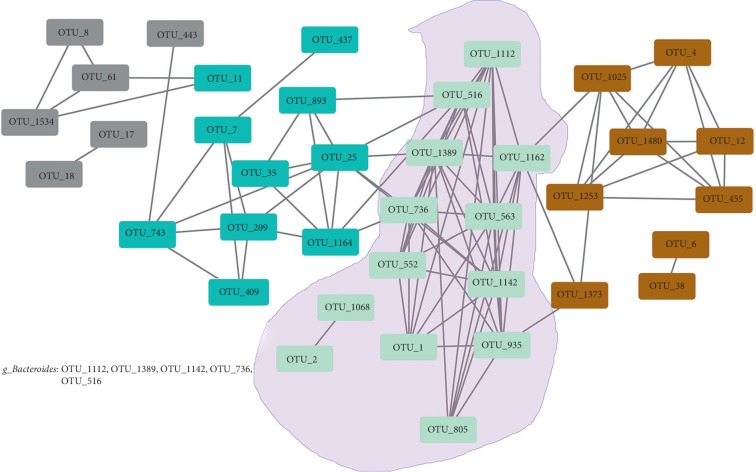
Network obtained by WGCNA. In this graph, the isolated nodes are removed from the network. Four modules, named MEgrey, MEturquoise, MEblue, and MEbrown, include 6, 13, 10, and 9 OTUs, respectively. The important module, MEturquoise, is highlighted with purple shadows. In MEturquoise, OTU_1112, OTU_1389, OTU_1142, OTU_736, and OTU_516 all represent *g_Bacteroides*.

After the edge weights were filtered according to *r* > 0.1, a simplified network was attained. The size of the network, the number of network edges, the average degree, the average path length, and the graph density were 38, 94, 4.947, 2.891, and 0.134, respectively. Hence, we extracted the top 10 OTUs with outstanding topological properties (Table 2). These OTUs had higher degree, betweenness, and closeness demonstrating that these OTUs link with further OTUs, and thus, potential OTUs were associated with GO. As shown in Table 2, the majority of these OTUs (90%) were contained in the turquoise module (MEturquoise). Module preservation was assessed using the Z-summary, and the results showed that conservation of a turquoise module was confirmed. The simplified network obtained from WGCNA is illustrated in Figure 4. In this network, the isolated nodes were removed. From Table 2 and Figure 4, we can see that the proportion of Bacteroidetes in the top ten OTUs is very high.

### 3.3. Correlation between GO-Related Traits and Gut Microbiota

#### 3.3.1. Correlation between Modules and GO-Related Traits

**Figure 5:**
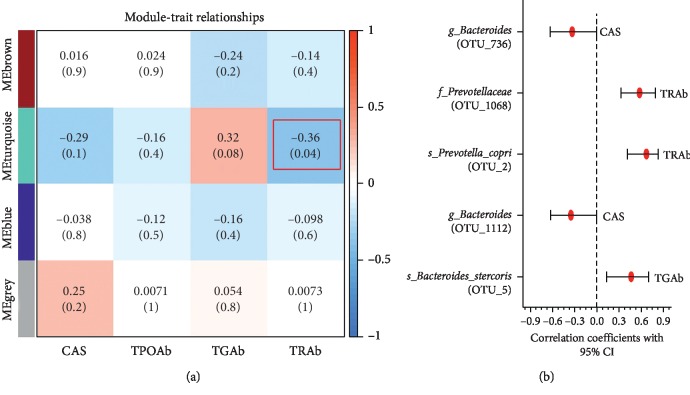
Association between the modules and GO-related traits. (a) Correlation analysis between four modules and GO-related traits. (b) Significant association between each of the bacteria involved in MEturquoise and GO-related traits. CI: confidence interval.

Pearson's correlation analysis was performed between the identified modules and GO-related traits, in which the traits were CAS, TRAb, TPOAb, and TGAb. The first principle component of a module was used to calculate Pearson's correlation coefficient between a module and a GO-related trait. We noted that the turquoise module (MEturquoise), which contained the majority of the OTUs with outstanding topological properties, was negatively correlated with TRAb (*r* = −0.36, *P*=0.04) (Figure 5(a)). As depicted in Figure 5(a), each cell of the matrix contained a correlation coefficient between one OTU module and a GO-related trait.

#### 3.3.2. Correlation between OTUs Involved in MEturquoise and GO-Related Traits

We further performed Pearson's correlation analysis between OTUs involved in the turquoise module (MEturquoise) and GO-related traits, and the traits included CAS, TRAb, TPOAb, and TGAb. We found only five bacteria: *s_Prevotella_copri* (OTU_2), *s_Bacteroides_stercoris* (OTU_5), *g_Bacteroides* (OTU_736), *f_Prevotellaceae* (OTU_1068), and *g_Bacteroides* (OTU_1112), which showed a significant correlation with GO-related traits, such as CAS, TRAb, and TGAb (Figure 5(b)).

### 3.4. Negative Binomial Regression Model to Find Out the Relationships between GO-Related Traits and Gut Microbiota

As the five identified bacteria showed a significant association with GO-related traits, we additionally used the negative binomial regression model to investigate the relationships between these bacteria and GO-related traits (TRAb, CAS, and TGAb) after adjusting for age and sex. As presented in Table 3, *s_Prevotella_copri*, *s_Bacteroides_stercoris*, and *f_Prevotellaceae* were all correlated with TRAb and TGAb, while *g_Bacteroides* was correlated with CAS.

### 3.5. LCA to Classify GO Patients

**Figure 6:**
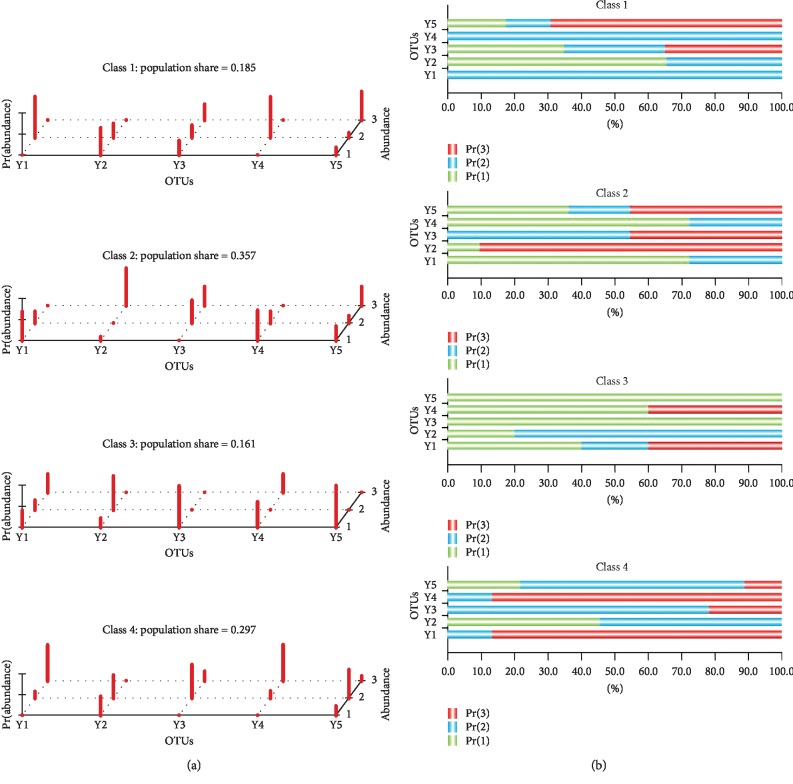
Latent variable analysis based on five bacteria. (a) 3D plot of the latent model. In the OTU axis, Y1, Y2, Y3, Y4, and Y5 indicate five bacteria: *s_Prevotella_copri*, *s_Bacteroides_stercoris*, *f_Prevotellaceae*, *g_Bacteroides* (OTU_736), and *g_Bacteroides* (OTU_1112). In the abundance axis, 1, 2, and 3 indicate three levels of absolute abundance (<33rd percentile, >33rd percentile and <66rd percentile, and >66rd percentile), respectively. In the Pr(abundance) axis, the red bar indicates the posterior probability of five bacteria responses across the classes. (b) 2D plot of the latent model showing the same information as that of the 3D plot.

In this stage, the five identified bacteria (*s_Prevotella_copri* (Y1), *s_Bacteroides_stercoris* (Y2), *f_Prevotellaceae* (Y3), *g_Bacteroides* (Y4, OTU_736), and *g_Bacteroides* (Y5, OTU_1112)), which showed the significant correlation with GO-related traits, were selected. In LCA, the absolute abundance of the five bacteria was divided into three levels (<33rd percentile, >33rd percentile and <66th percentile, and >66th percentile) and were then coded as 1, 2, and 3. We applied LCA to detect the presence of the bacteria in latent classes. After applying multiple evaluation indexes in LCA (Supplementary [Supplementary-material supplementary-material-1] and Supplementary [Supplementary-material supplementary-material-1]), four latent variables were set; besides, the results of analysis are shown in Table 4 and Figure 6.

**Figure 7:**
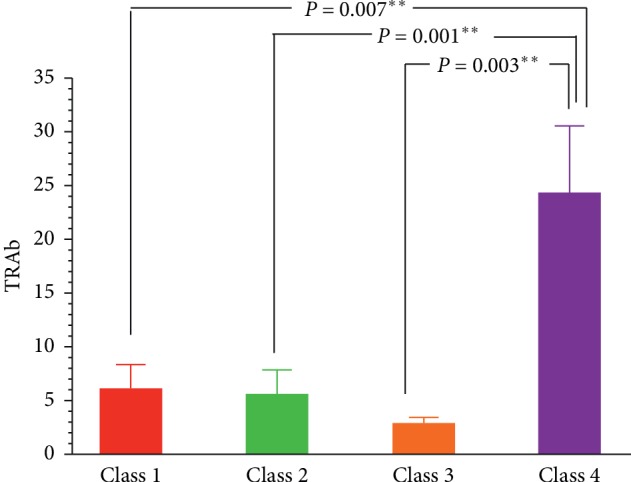
Comparison of the difference of TRAb among the four latent classes. The fourth latent class has a higher TRAb value than other three latent classes. The fourth latent class included GO patients with high abundance of *s_Prevotella_copri* and *g_Bacteroides*. ^*∗∗*^*P* < 0.01.

We then compared differences in TRAb among the four latent classes. The results showed that the fourth latent class had a higher TRAb value than other three latent classes. The fourth latent class included GO patients with high abundance of *s_Prevotella_copri* and *g_Bacteroides* (OTU_736) (Figure 7).

## 4. Discussion

In the present study, we applied a metabolic-network-driven analysis to explore the relationships between gut microbiota and GO-related traits and identified a number of novel associations between gut microbiota and serum TRAb. In our previous study, linear discriminant analysis effect size (LEfSe) analysis and random forest analysis showed that Prevotellaceae was one discriminative feature, which could distinguish GO patients from controls obviously [9]. In the present study, Prevotellaceae was identified as an important family of bacteria associated with TRAb. Additionally, we applied LCA to classify GO patients into four different subclasses based on their gut microbiota constitution and found that those GO patients with high abundance of *s_Prevotella_copri* and *g_Bacteroides* had a higher TRAb level.

In humans, approximately 30–400 trillion microorganisms colonize the human intestinal tract, and their composition depends on environmental and immunogenetic factors [18]. Alterations in bacterial function and diversity may contribute to the development of autoimmune diseases and infectious diseases [19]. A number of scholars demonstrated that intestinal dendritic cells and macrophages are hyperresponsive to pathogen‐associated molecular patterns during equilibrium. When epithelial barrier breakdown occurs, the pattern recognition receptors, which are present in innate immune cells, detect gut microbiota through toll‐like receptors, soluble retinoic acid‐inducible gene I, NOD‐like receptors, or melanoma differentiation‐associated protein 5, inducing an inflammatory cascade and activation of adaptive immune responses [20, 21]. The variations in gut microbiota composition have been described in patients suffering from autoimmune diseases, including GD and HT [22–24]. A previous research demonstrated that the gut is mainly inhabited by two phyla of bacteria in humans: Firmicutes and Bacteroidetes, and the latter was mostly dominated by *Bacteroides* and *Prevotella* genera [25]. Studies showed that *Bacteroides* bacteria play an important role in consuming carbohydrates, while the *Prevotella* species dominate fibers especially [26, 27]. A number of scholars reported that *Prevotella* is associated with chronic inflammatory conditions, including rheumatoid arthritis (RA) and systemic T-cell activation in HIV-1 infection [28, 29]. In addition, *s_Prevotella_copri*, the most abundant species in *Prevotella*, has been found to be correlated with the development of rheumatoid arthritis (RA). It has been previously unveiled that patients with RA have differential reactivity of immunoglobulin G (IgG) or IgA immune responses with *s_Prevotella_copri*. The responses were either IgG antibodies to *P*. *copri*, suggestive of a systemic immune response, or IgA antibody responses, suggestive of a mucosal immune response [30]. To date, the correlation of *s_Prevotella_copri* with thyroid-associated diseases has remained elusive. In the present study, the level of *s_Prevotella_copri* was positively correlated with a higher serum level of TRAb, which may be related to active orbital inflammation. In the current research, we found that *f_Prevotellaceae* was correlated with levels of TRAb in GO patients. Next, we detected *f_Prevotellaceae* DNA in the gut and antibody in the serum in patients [31]. The study showed that patients with inflammatory bowel disease (IBD) may exhibit a concomitant increase in Bacteroidetes [32]. In the present study, patients with higher serum levels of TRAb had high abundance of *s_Prevotella_copri* and *g_Bacteroides* in LCA. As expressed previously, TRAb is an independent risk factor for GO and can predict severity and outcome of the disease [33]. The present research revealed the relationship between bacteria and TRAb, and our findings may be helpful for the prediction of GO by intestinal microorganisms in the future study. The relationship between *s_Prevotella_copri* and *g_Bacteroides* in regulating TRAb-associated immune responses needs to be further explored and elucidated.

The gut microbiome is a complex and metabolically active community that directly influences host phenotypes [34]. In fact, the structure of the gut microbiome is influenced by several factors, including interactions between its members. Therefore, it is highly essential to understand the combination of microbial effects underlying their contributions to disease [35]. A general approach to infer interactions between bacteria in the gut microbiota is to quantify the cooccurrence of OTUs. To address this issue, network biology is an emerging field that represents biology as networks that capture the relations between the parts of a complex biological system, such as molecules, processes, organs, or even different organisms. Meanwhile, WGCNA, which is used to identify modules in gene coexpression networks, can facilitate identification of communities within microbial cooccurrence networks [34, 36]. For instance, a study showed that certain metabolites strongly correlate with the microbial community structure, and some of these correlations are specific for the prediabetic state [34]. In the present study, we assessed the association between gut microbiota and GO-related traits in the combination of microbial effects.

The present study has a number of limitations: First, the limited sample size influenced the achieved results. To address this issue, we performed the data dimensionality reduction. In WGCNA, we kept those OTUs found in at least 30% of the samples in order to ensure that the data were less sparse, as well as being more amenable to calculate correlation coefficients; besides, we referenced a previous study for preselecting bacteria. In addition, we used module preservation analysis to estimate the robustness of the identified modules and analyzed the topological properties to identify key bacteria. Specially, we applied the correlation analysis, negative binomial regression, and LCA to further validate the identified bacteria. Although these layer-by-layer analyses achieved the goal of the data dimensionality reduction and avoided the identified bacteria to be false-positive, an enlarged sample size will be required in the future study to validate the potential relationship between these bacteria and GO. Second, whether the changes in the fecal microbiota might be a cause or a consequence of GO development should be further explored. The analysis of 16S rRNA gene fragments did not provide data related to the functional traits of the bacterial genera being present in the communities. In addition, Pearson and Spearman's correlations were limited to detect the cooccurrence of bacteria; thus, further ensemble approaches, such as SparCC, Kullback–Leibler divergence, and Bray–Curtis dissimilarity, will be used in the next study. Third, our findings may be related to thyroid autoimmunity rather than GO; thus, further analyses about the gut bacteria between GO patients and patients with GD without GO should be performed. Finally, TRAbs were subdivided into thyroid-stimulating antibody (TSAb), thyroid-blocking antibody (TBAb), and neutral antibody (neutral Ab) according to their functional effects [10]. The assay did not distinguish between stimulating, blocking, and no functional effects on the thyroid gland.

In summary, our study suggested the potential relationships between the composition of the gut microbiota and TRAb in GO patients. It might provide a new insight into the interaction between the intestinal microbiota and TRAb-associated immune responses in GO patients.

## Figures and Tables

**Table 1:** Clinical characteristics of GO subjects.

	Sex (M/F)	Age (years)	Height (cm)	Weight (kg)	GO duration (months)	CAS	NOSPECS	FT3 (pmol/L)	FT4 (pmol/L)	T3 (pmol/L)	T4 (pmol/L)	TSH (uIU/ml)	TPOAb (IU/ml)	TGAb (IU/ml)	TRAb (IU/L)
Case 31	15/16	45.4 ± 11.8	167.5 ± 9.2	66.8 ± 10.9	7.2 ± 4.1	3.2 ± 0.6	IV (29) V (2)	5.36 (4.81, 6.67)	16.18 (12.81, 19.48)	1.91^*∗*^ (1.74, 2.33)	107.10 (85.40, 124.10)	0.42^*∗*^ (0.05, 1.54)	13.36 (7.81, 134.28)	14.72^*∗*^ (10.13, 79.39)	3.68^*∗*^ (1.42–14.98)

Values are mean (SD), median (range), or *n* (%). Comparison between any two groups by Mann–Whitney *U* test, ^*∗*^*P* < 0.05. SBP: systolic blood pressure; DBP: diastolic blood pressure; CAS: clinical activity score; TSH: thyroid-stimulating hormone; TPOAb: thyroperoxidase antibody; TGAb: antithyroglobulin antibody; TRAb: thyrotropin receptor antibody.

**Table 2:** The top ten OTU taxa with outstanding topological properties.

OTU number	Name	Degree	Betweenness	Closeness	Module
OTU_1389	*g_Bacteroides*	11	57.807	0.085	MEturquoise
OTU_1142	*g_Bacteroides*	11	57.807	0.085	MEturquoise
OTU_736	*g_Bacteroides*	10	26.117	0.084	MEturquoise
OTU_563	*s_Bacteroides_vulgatus*	10	10.797	0.084	MEturquoise
OTU_1112	*g_Bacteroides*	10	10.797	0.084	MEturquoise
OTU_935	*g_Faecalibacterium*	9	23.552	0.084	MEturquoise
OTU_516	*g_Bacteroides*	9	18.28	0.083	MEturquoise
OTU_25	*s_Bacteroides_ovatus*	9	132.281	0.084	MEblue
OTU_1162	*g_Faecalibacterium*	8	121.202	0.084	MEturquoise
OTU_552	*g_Bacteroides_vulgatus*	7	0.267	0.082	MEturquoise

OTU: operational taxonomic unit.

**Table 3:** Associations between gut microbiota and GO-related traits.

Gut microbiota	TRAb		TGAb		CAS	
	*β*	*P*	*β*	*P*	*β*	*P*
*s_Prevotella_copri* (OTU_2)	0.112	<0.001^*∗∗*^	−0.003	<0.001^*∗∗*^	0.060	0.946
*s_Bacteroides_stercoris* (OTU_5)	−0.066	0.006^*∗∗*^	−0.0003	<0.001^*∗∗*^	−0.215	0.619
*g_Bacteroides* (OTU_736)	−0.008	0.599	−0.0004	0.209	−1.225	0.002^*∗∗*^
*f_Prevotellaceae* (OTU_1068)	0.056	0.011^*∗*^	−0.0002	0.006^*∗∗*^	0.445	0.638
*g_Bacteroides* (OTU_1112)	−0.019	0.303	−0.0005	0.322	−1.286	<0.001^*∗∗*^

Negative binomial regressions were performed on gut microbiota after adjusting for age and sex. ^*∗*^*P* < 0.05; ^*∗∗*^*P* < 0.01. CAS: clinical activity score; TGAb: antithyroglobulin antibody; TRAb: thyrotropin receptor antibody.

**Table 4:** Latent variable analysis based on the five identified bacteria.

	latent1	latent2	latent3	latent4
Class probability^a^	0.1847	0.3567	0.1613	0.2973

Y1 (OTU_2, *s_Prevotella_copri*)				
1	0.0000	0.7234	0.4000	0.0000
2	1.0000	0.2766	0.2000	0.1319
3	0.0000	0.0000	0.4000	0.8681

Y2 (OTU_5, *s_Bacteroides_stercoris*)				
1	0.6546	0.0958	0.2000	0.4550
2	0.3454	0.0000	0.8000	0.5450
3	0.0000	0.9042	0.0000	0.0000

Y3 (OTU_1068, *f_Prevotellaceae*)				
1	0.3493	0.0000	1.0000	0.0000
2	0.3056	0.5462	0.0000	0.7823
3	0.3450	0.4538	0.0000	0.2177

Y4 (OTU_736, *g_Bacteroides*)				
1	0.0000	0.7234	0.6000	0.0000
2	1.0000	0.2766	0.0000	0.1319
3	0.0000	0.0000	0.4000	0.8681

Y5 (OTU_1112, *g_Bacteroides*)				
1	0.1747	0.3617	1.0000	0.2170
2	0.1341	0.1845	0.0000	0.6718
3	0.6912	0.4538	0.0000	0.1111

^a^1, 2, and 3 indicate three levels of absolute abundance (<33rd percentile, >33rd percentile and <66rd percentile, and >66rd percentile) of each bacterium.

## Data Availability

The data that support the findings of this study are available from the corresponding author upon reasonable request.

## Abbreviations

GO: Graves' orbitopathy
GD: Graves' disease
T1D: Type 1 diabetes
SLE: Systemic lupus erythematosus
HT: Hashimoto's thyroiditis
TRAb: Thyrotropin receptor autoantibody
OTUs: Operational taxonomic units
CAS: Clinical activity score
TPOAb: Thyroperoxidase antibody
TGAb: Antithyroglobulin antibody
WGCNA: Weighted gene coexpression network analysis
LCA: Latent class analysis
LR: Likelihood ratio
TSAb: Thyroid-stimulating antibody
TBAb: Thyroid-blocking antibody
IBD: Inflammatory bowel disease.

## Supplementary Materials

Supplementary Materials Supplementary Table 1: the corresponding OTUs of the simplified network obtained from WGCNA. Supplementary Table 2: multiple evaluation indexes of LCA. The evaluation indexes of LCA are cAIC, aBIC, entropy, and likelihood ratio (LR). The smaller the LR, the smaller the AIC and the smaller the BIC, which means better fitting of the data. The greater the entropy, the better the model fits the data. Supplementary Figure 1: soft thresholding of power. In WGCNA, scale independence and mean connectivity were used for power selection. When power = 1, the fitted *R*^2^ value and the mean connectivity are all the highest. Supplementary Figure 2: multiple evaluation indexes of LCA. The evaluation indexes of LCA are cAIC, aBIC, entropy, and likelihood ratio (LR). The smaller the LR, the smaller the AIC and the smaller the BIC, which means better fitting of the data. The greater the entropy, the better the model fits the data.

## References

[1] Kahaly G. J., Bartalena L., Hegedüs L., Leenhardt L., Poppe K., Pearce S. H. (2018). 2018 European thyroid association guideline for the management of Graves’ hyperthyroidism. *European Thyroid Journal*.

[2] Bartalena L., Baldeschi L., Boboridis K. (2016). The 2016 European thyroid association/European group on Graves’ orbitopathy guidelines for the management of Graves’ orbitopathy. *European Thyroid Journal*.

[3] Drui D., Du Pasquier Fediaevski L., Vignal Clermont C., Daumerie C. (2018). Graves’ orbitopathy: diagnosis and treatment. *Annales d’Endocrinologie*.

[4] Kugelberg E. (2017). Diet can protect against type 1 diabetes. *Nature Reviews Immunology*.

[5] Edwards C., Costenbader K. (2014). Epigenetics and the microbiome: developing areas in the understanding of the aetiology of lupus. *Lupus*.

[6] Mori K., Nakagawa Y., Ozaki H. (2012). Does the gut microbiota trigger Hashimoto’s thyroiditis?. *Discovery medicine*.

[7] Manicassamy S., Ravindran R., Deng J. (2009). Toll-like receptor 2-dependent induction of vitamin A-metabolizing enzymes in dendritic cells promotes T regulatory responses and inhibits autoimmunity. *Nature Medicine*.

[8] Sasso F. C., Carbonara O., Torella R. (2004). Ultrastructural changes in enterocytes in subjects with Hashimoto’s thyroiditis. *Gut*.

[9] Shi T. T., Xin Z., Hua L. (2019). Alterations in the intestinal microbiota of patients with severe and active Graves’ orbitopathy: a cross-sectional study. *Journal of Endocrinological Investigation*.

[10] Seo S., Sánchez Robledo M. (2018). Usefulness of TSH receptor antibodies as biomarkers for Graves’ ophthalmopathy: a systematic review. *Journal of Endocrinological Investigation*.

[11] Bluszcz G. A., Bednarczuk T., Bartoszewicz Z. (2018). Clinical utility of TSH receptor antibody levels in Graves’ orbitopathy: a comparison of two TSH receptor antibody immunoassays. *Central European Journal of Immunology*.

[12] Iyer S., Bahn R. (2012). Immunopathogenesis of Graves’ ophthalmopathy: the role of the TSH receptor. *Best Practice & Research Clinical Endocrinology & Metabolism*.

[13] Kahaly G. J., Wuster C., Olivo P. D., Diana T. (2019). High titers of thyrotropin receptor antibodies are associated with orbitopathy in patients with Graves’ disease. *The Journal of Clinical Endocrinology & Metabolism*.

[14] Zhou Y., Ou Z., Tang X. (2018). Alterations in the gut microbiota of patients with acquired immune deficiency syndrome. *Journal of Cellular and Molecular Medicine*.

[15] Bauer E., Thiele I. (2018). From network analysis to functional metabolic modeling of the human gut microbiota. *mSystems*.

[16] Callahan B. J., McMurdie P. J., Holmes S. P. (2017). Exact sequence variants should replace operational taxonomic units in marker-gene data analysis. *The ISME Journal*.

[17] Sankaran K., Holmes S. P. (2018). Latent variable modeling for the microbiome. *Biostatistics*.

[18] Kamada N., Seo S.-U., Chen G. Y., Núñez G. (2013). Role of the gut microbiota in immunity and inflammatory disease. *Nature Reviews Immunology*.

[19] Kamada N., Núñez G. (2013). Role of the gut microbiota in the development and function of lymphoid cells. *The Journal of Immunology*.

[20] de Oliveira G. L. V., Leite A. Z., Higuchi B. S., Gonzaga M. I., Mariano V. S. (2017). Intestinal dysbiosis and probiotic applications in autoimmune diseases. *Immunology*.

[21] Bevins C. L., Salzman N. H. (2011). Paneth cells, antimicrobial peptides and maintenance of intestinal homeostasis. *Nature Reviews Microbiology*.

[22] Virili C., Fallahi P., Antonelli A., Benvenga S., Centanni M. (2018). Gut microbiota and Hashimoto’s thyroiditis. *Reviews in Endocrine and Metabolic Disorders*.

[23] Köhling H. L., Plummer S. F., Marchesi J. R., Davidge K. S., Ludgate M. (2017). The microbiota and autoimmunity: their role in thyroid autoimmune diseases. *Clinical Immunology*.

[24] Zhao F., Feng J., Li J. (2018). Alterations of the gut microbiota in hashimoto’s thyroiditis patients. *Thyroid*.

[25] Rodriguez J. M., Murphy K., Stanton C. (2015). The composition of the gut microbiota throughout life, with an emphasis on early life. *Microbial Ecology in Health & Disease*.

[26] Wexler H. M. (2007). Bacteroides: the good, the bad, and the nitty-gritty. *Clinical Microbiology Reviews*.

[27] Wu G. D., Chen J., Hoffmann C. (2011). Linking long-term dietary patterns with gut microbial enterotypes. *Science*.

[28] Larsen J. M. (2017). The immune response to *Prevotella* bacteria in chronic inflammatory disease. *Immunology*.

[29] Armstrong A. J. S., Shaffer M., Nusbacher N. M. (2018). An exploration of *Prevotella*-rich microbiomes in HIV and men who have sex with men. *Microbiome*.

[30] Pianta A., Arvikar S., Strle K. (2017). Evidence of the immune relevance of *Prevotella copri* , a gut microbe, in patients with rheumatoid arthritis. *Arthritis & Rheumatology*.

[31] Rosenberg E., Rosenberg E., DeLong E. F., Lory S., Stackebrandt E., Thompson F. (2014). The family *Prevotellaceae*. *The Prokaryotes: Other Major Lineages of Bacteria and the Archaea*.

[32] Hansen J., Gulati A., Sartor R. B. (2010). The role of mucosal immunity and host genetics in defining intestinal commensal bacteria. *Current Opinion in Gastroenterology*.

[33] Eckstein A. K., Plicht M., Lax H. (2006). Thyrotropin receptor autoantibodies are independent risk factors for Graves’ ophthalmopathy and help to predict severity and outcome of the disease. *The Journal of Clinical Endocrinology & Metabolism*.

[34] Org E., Blum Y., Kasela S. (2017). Relationships between gut microbiota, plasma metabolites, and metabolic syndrome traits in the METSIM cohort. *Genome Biology*.

[35] Faust K., Raes J. (2012). Microbial interactions: from networks to models. *Nature Reviews Microbiology*.

[36] Langfelder P., Horvath S. (2008). WGCNA: an *R* package for weighted correlation network analysis. *BMC Bioinformatics*.

