# Supplementary material for: The Potential Link between Gut Microbiota and Serum TRAb in Chinese Patients with Severe and Active Graves' Orbitopathy
Source: Int J Endocrinol. 2019 Dec 18;2019:9736968. doi: 10.1155/2019/9736968 (PMC6942819; doi:10.1155/2019/9736968)

**Supplementary Table 1 The corresponding OTUs of simplified network obtained from WGCNA analysis.**

| #OTU_num | Taxonomy |
| --- | --- |
| OTU_1 | k__Bacteria;p__Bacteroidetes;c__Bacteroidia;o__Bacteroidales;f__Bacteroidaceae;g__Bacteroides |
| OTU_2 | k__Bacteria;p__Bacteroidetes;c__Bacteroidia;o__Bacteroidales;f__Prevotellaceae;g__unidentified_Prevotellaceae;s__Prevotella_copri |
| OTU_4 | k__Bacteria;p__Firmicutes;c__Clostridia;o__Clostridiales;f__Ruminococcaceae;g__Faecalibacterium;s__ |
| OTU_6 | k__Bacteria;p__Firmicutes;c__Clostridia;o__Clostridiales;f__Ruminococcaceae;g__Subdoligranulum;s__ |
| OTU_7 | k__Bacteria;p__Bacteroidetes;c__Bacteroidia;o__Bacteroidales;f__Bacteroidaceae;g__Bacteroides;s__Bacteroides_uniformis |
| OTU_8 | k__Bacteria;p__Firmicutes;c__Clostridia;o__Clostridiales;f__Ruminococcaceae;g__Subdoligranulum;s__ |
| OTU_11 | k__Bacteria;p__Bacteroidetes;c__Bacteroidia;o__Bacteroidales;f__Bacteroidaceae;g__Bacteroides;s__Bacteroides_fragilis |
| OTU_12 | k__Bacteria;p__Actinobacteria;c__unidentified_Actinobacteria;o__Bifidobacteriales;f__Bifidobacteriaceae;g__Bifidobacterium;s__Bifidobacterium_adolescentis |
| OTU_17 | k__Bacteria;p__Bacteroidetes;c__Bacteroidia;o__Bacteroidales;f__Bacteroidaceae;g__Bacteroides;s__Bacteroides_caccae |
| OTU_18 | k__Bacteria;p__Firmicutes;c__Erysipelotrichia;o__Erysipelotrichales;f__Erysipelotrichaceae;g__unidentified_Erysipelotrichaceae;s__bacterium_LF-3 |
| OTU_25 | k__Bacteria;p__Bacteroidetes;c__Bacteroidia;o__Bacteroidales;f__Bacteroidaceae;g__Bacteroides;s__Bacteroides_ovatus |
| OTU_35 | k__Bacteria;p__Firmicutes;c__Clostridia;o__Clostridiales;f__Lachnospiraceae;g__Lachnoclostridium;s__ |
| OTU_38 | k__Bacteria;p__Firmicutes;c__Clostridia;o__Clostridiales;f__Lachnospiraceae |
| OTU_61 | k__Bacteria;p__Firmicutes;c__Clostridia;o__Clostridiales;f__Lachnospiraceae;g__unidentified_Lachnospiraceae;s__butyrate-producing_bacterium_GM2/1 |
| OTU_209 | k__Bacteria;p__Bacteroidetes;c__Bacteroidia;o__Bacteroidales;f__Bacteroidaceae;g__Bacteroides;s__Bacteroides_thetaiotaomicron |
| OTU_409 | k__Bacteria;p__Bacteroidetes;c__Bacteroidia;o__Bacteroidales;f__Bacteroidaceae;g__Bacteroides |
| OTU_437 | k__Bacteria;p__Bacteroidetes;c__Bacteroidia;o__Bacteroidales;f__Bacteroidaceae;g__Bacteroides;s__Bacteroides_uniformis |
| OTU_443 | k__Bacteria;p__Bacteroidetes;c__Bacteroidia;o__Bacteroidales;f__Tannerellaceae;g__Parabacteroides |
| OTU_455 | k__Bacteria;p__Firmicutes;c__Clostridia;o__Clostridiales;f__Ruminococcaceae;g__Faecalibacterium |
| OTU_516 | k__Bacteria;p__Bacteroidetes;c__Bacteroidia;o__Bacteroidales;f__Bacteroidaceae;g__Bacteroides |
| OTU_552 | k__Bacteria;p__Bacteroidetes;c__Bacteroidia;o__Bacteroidales;f__Bacteroidaceae;g__Bacteroides;s__Bacteroides_vulgatus |
| OTU_563 | k__Bacteria;p__Bacteroidetes;c__Bacteroidia;o__Bacteroidales;f__Bacteroidaceae;g__Bacteroides;s__Bacteroides_vulgatus |
| OTU_736 | k__Bacteria;p__Bacteroidetes;c__Bacteroidia;o__Bacteroidales;f__Bacteroidaceae;g__Bacteroides |
| OTU_743 | k__Bacteria;p__Bacteroidetes;c__Bacteroidia;o__Bacteroidales;f__Bacteroidaceae;g__Bacteroides;s__Bacteroides_thetaiotaomicron |
| OTU_805 | k__Bacteria;p__Firmicutes;c__Clostridia;o__Clostridiales;f__Ruminococcaceae;g__Faecalibacterium |
| OTU_893 | k__Bacteria;p__Bacteroidetes;c__Bacteroidia;o__Bacteroidales;f__Tannerellaceae;g__Parabacteroides |
| OTU_935 | k__Bacteria;p__Firmicutes;c__Clostridia;o__Clostridiales;f__Ruminococcaceae;g__Faecalibacterium;s__ |
| OTU_1025 | k__Bacteria;p__Firmicutes;c__Clostridia;o__Clostridiales;f__Ruminococcaceae;g__Faecalibacterium |
| OTU_1068 | k__Bacteria;p__Bacteroidetes;c__Bacteroidia;o__Bacteroidales;f__Prevotellaceae |
| OTU_1112 | k__Bacteria;p__Bacteroidetes;c__Bacteroidia;o__Bacteroidales;f__Bacteroidaceae;g__Bacteroides |
| OTU_1142 | k__Bacteria;p__Bacteroidetes;c__Bacteroidia;o__Bacteroidales;f__Bacteroidaceae;g__Bacteroides |
| OTU_1162 | k__Bacteria;p__Firmicutes;c__Clostridia;o__Clostridiales;f__Ruminococcaceae;g__Faecalibacterium |
| OTU_1164 | k__Bacteria;p__Bacteroidetes;c__Bacteroidia;o__Bacteroidales;f__Tannerellaceae;g__Parabacteroides |
| OTU_1253 | k__Bacteria;p__Firmicutes;c__Clostridia;o__Clostridiales;f__Ruminococcaceae;g__Faecalibacterium |
| OTU_1373 | k__Bacteria;p__Firmicutes;c__Clostridia;o__Clostridiales;f__Ruminococcaceae;g__Faecalibacterium |
| OTU_1389 | k__Bacteria;p__Bacteroidetes;c__Bacteroidia;o__Bacteroidales;f__Bacteroidaceae;g__Bacteroides |
| OTU_1480 | k__Bacteria;p__Firmicutes;c__Clostridia;o__Clostridiales;f__Ruminococcaceae;g__Faecalibacterium;s__Faecalibacterium_prausnitzii |
| OTU_1534 | k__Bacteria;p__Bacteroidetes;c__Bacteroidia;o__Bacteroidales;f__Bacteroidaceae;g__Bacteroides |

**Supplementary Table 2 The multiple evaluation indexes of LCA analysis.** The evaluation indexes of LCA are cAIC, aBIC, Entropy and the Likelihood-ratio (LR). The smaller the LR, the smaller AIC and the smaller BIC means the better fitting the data. The greater the Entropy, the better the model fits the data.

| The number of class | Log-likelihood | BIC | aBIC | cAIC | Likelihood-ratio | Entropy |
| --- | --- | --- | --- | --- | --- | --- |
| 2 | -143.93 | 359.98 | 294.56 | 380.98 | 91.60 | 0.96 |
| 3 | -135.88 | 381.65 | 281.95 | 413.65 | 75.49 | 0.98 |
| 4 | -128.13 | 403.92 | 269.95 | 446.92 | 59.99 | 0.98 |
| 5 | -121.24 | 427.91 | 259.67 | 481.91 | 46.20 | 0.99 |

**Supplementary Figure 1 The soft thresholding of power.** In WGCNA analysis, Scale independence and Mean connectivity were used to power selection. When power=1, the fitted R^2^ value and the mean connectivity are all the highest.

**
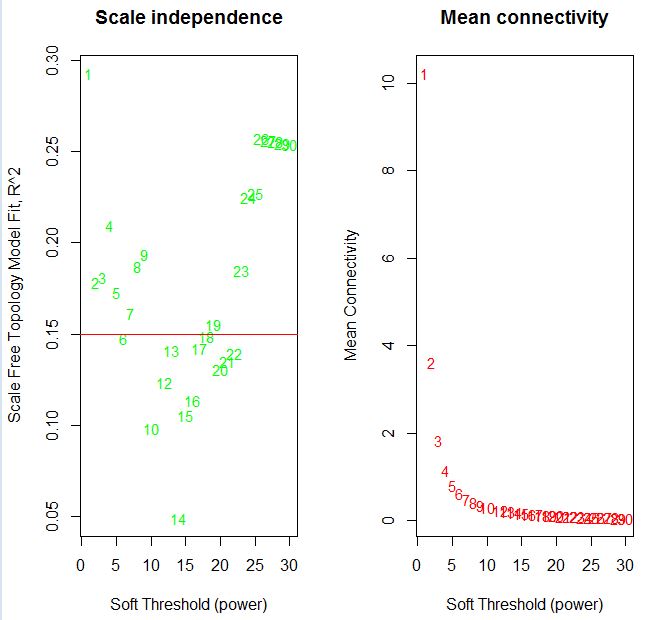
**

**Supplementary Figure 2 The multiple evaluation indexes of LCA analysis.** The evaluation indexes of LCA are cAIC, aBIC, Entropy and the Likelihood-ratio (LR). The smaller the LR, the smaller AIC and the smaller BIC means the better fitting the data. The greater the Entropy, the better the model fits the data.


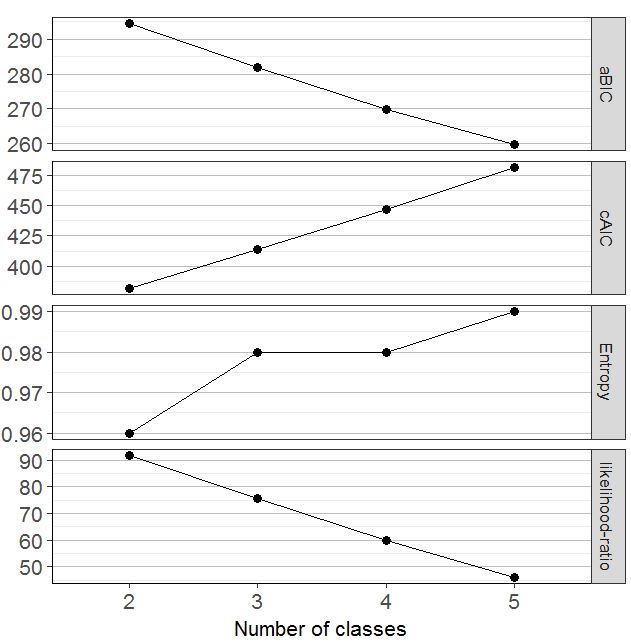

Supplement: Supplementary Materials — Supplementary Table 1: the corresponding OTUs of the simplified network obtained from WGCNA. Supplementary Table 2: multiple evaluation indexes of LCA. The evaluation indexes of LCA are cAIC, aBIC, entropy, and likelihood ratio (LR). The smaller the LR, the smaller the AIC and the smaller the BIC, which means better fitting of the data. The greater the entropy, the better the model fits the data. Supplementary Figure 1: soft thresholding of power. In WGCNA, scale independence and mean connectivity were used for power selection. When power = 1, the fitted R2 value and the mean connectivity are all the highest. Supplementary Figure 2: multiple evaluation indexes of LCA. The evaluation indexes of LCA are cAIC, aBIC, entropy, and likelihood ratio (LR). The smaller the LR, the smaller the AIC and the smaller the BIC, which means better fitting of the data. The greater the entropy, the better the model fits the data. [file 9736968.f1.docx]
